# Tax as a therapeutic target in ATL

**DOI:** 10.1186/1742-4690-12-S1-O20

**Published:** 2015-08-28

**Authors:** Zeina Dassouki, Umut Sahin, Hiba El Hajj, Florence Jollivet, Youmna Kfoury, Valérie Lallemand-Breitenbach, Olivier Hermine, Hugues de The, Ali Bazarbachi

**Affiliations:** 1 Department of Internal Medicine, Faculty of Medicine, American University of Beirut, Beirut, Lebanon; 2 Université Paris Diderot, Sorbonne Paris Cité, Hôpital St. Louis 1, Avenue Claude Vellefaux 75475 PARIS cedex 10, France; 3 INSERM UMR 944, Equipe labellisée par la Ligue Nationale contre le Cancer, Institut Universitaire d'Hématologie, Hôpital St. Louis 1, Avenue Claude Vellefaux 75475 PARIS cedex 10, France; 4 CNRS UMR 7212, Hôpital St. Louis 1, Avenue Claude Vellefaux 75475 PARIS cedex 10 France; 5 AP-HP, Service de Biochimie, Hôpital St. Louis 1, Avenue Claude Vellefaux 75475 PARIS cedex 10, France; 6 College de France, Place Marcelin Berthelot 75005 PARIS France; 7 CNRS UMR 8147, Hôpital Necker, PARIS cedex 15, France

## 

The HTLV-1 Tax transactivator initiates transformation in adult T-cell leukemia/lymphoma (ATL), a highly aggressive chemotherapy-resistant malignancy. The arsenic/interferon combination, which triggers degradation of the Tax on coprotein, selectively induces apoptosis of ATL cell lines and has significant clinical activity in Tax-driven murine ATL or patients. Yet, the role of Tax expression in maintaining the transformed phenotype and of Tax loss in ATL response is disputed and the molecular mechanisms driving degradation remain elusive. Here we demonstrate that ATL-derived or HTLV-1 transformed cells are addicted to continuous Tax expression, suggesting that Tax degradation underlies clinical responses to the arsenic/interferon combination. The latter enforces PML nuclear body (NB) formation and partner protein recruitment. In arsenic/interferon-treated ATL-derived cells, Tax is recruited onto NBs, undergoes PML-dependent hyper-sumoylation by SUMO2/3, but not SUMO1, ubiquitination by RNF4 and proteasome-dependent degradation. Thus, the arsenic/interferon combination clears ATL through degradation of its Tax driver and could have broader therapeutic value by promoting degradation of other pathogenic sumoylated proteins.

